# Initial Litter Chemistry and UV Radiation Drive Chemical Divergence in Litter during Decomposition

**DOI:** 10.3390/microorganisms12081535

**Published:** 2024-07-27

**Authors:** Bei Yao, Xiangshi Kong, Kai Tian, Xiaoyi Zeng, Wenshuo Lu, Lu Pang, Shucun Sun, Xingjun Tian

**Affiliations:** 1 School of Life Sciences, Nanjing University, Nanjing 210023, China; by-beiyao@hotmail.com (B.Y.); tiankai444422@163.com (K.T.); xyzeng@connect.hku.hk (X.Z.); hahalu9492@163.com (W.L.); panglugxs@163.com (L.P.); shcs@nju.edu.cn (S.S.); 2 Key Laboratory for Ecotourism of Hunan Province, School of Tourism, Jishou University, Jishou 416000, China; kongxiangshi@126.com; 3 Co-Innovation Center for Sustainable Forestry in Southern China, Nanjing Forestry University, Nanjing 210037, China

**Keywords:** litter decomposition, litter chemical complexity, initial litter chemistry, UV radiation, microbial communities, extracellular enzyme activity, ^13^C-CPMAS NMR

## Abstract

Litter’s chemical complexity influences carbon (C) cycling during its decomposition. However, the chemical and microbial mechanisms underlying the divergence or convergence of chemical complexity under UV radiation remain poorly understood. Here, we conducted a 397-day field experiment using ^13^C cross-polarization magic-angle spinning nuclear magnetic resonance (^13^C-CPMAS NMR) to investigate the interactions among the initial chemistry, microbial communities, and UV radiation during decomposition. Our study found that the initial concentrations of O-substituted aromatic C, di-O-alkyl C, and O-alkyl C in *Deschampsia caespitosa* were higher than those in *Kobresia tibetica*. Litter’s chemical composition exhibited divergent patterns based on the initial chemistry, UV radiation, and decay time. Specifically, *D. caespitosa* consistently displayed higher concentrations of di-O-alkyl C and O-alkyl C compared to *K. tibetica*, regardless of the UV exposure and decay time. Additionally, litter’s chemical complexity was positively correlated with changes in the extracellular enzyme activities, particularly those involved in lignin, cellulose, and hemicellulose degradation, which accounted for 9%, 20%, and 4% of the variation in litter’s chemical complexity, respectively. These findings highlighted the role of distinct microbial communities in decomposing different C components through catabolism, leading to chemical divergence in litter. During the early decomposition stages, oligotrophic Planctomycetes and Acidobacteria metabolized O-alkyl C and di-O-alkyl C under UV-blocking conditions. In contrast, copiotrophic Actinobacteria and Chytridiomycota utilized these components under UV radiation exposure, reflecting their ability to thrive under UV stress conditions due to their rapid growth strategies in environments rich in labile C. Our study revealed that the inherent differences in the initial O-alkyl C and di-O-alkyl C contributed to the chemical divergence, while UV radiation further influenced this divergence by shifting the microbial community composition from oligotrophic to copiotrophic species. Thus, differences in the initial litter chemistry, microbial community, and UV radiation affected the quantity and quality of plant-derived C during decomposition.

## 1. Introduction

Litter decomposition is a critical ecological process in terrestrial ecosystems, controlling the carbon (C) cycle and energy fluxes among the atmosphere, organisms, and soil [1]. This process is influenced by the interaction among environmental factors (such as climate and soil conditions), litter chemistry, and decomposers (including soil animals and microorganisms) [2,3,4,5]. The complexity of litter chemistry plays a crucial role in determining the turnover rate and sequestration pathways of organic C [6,7]. Current theories on the chemical complexity of organic C propose that variations in the initial litter chemistry and microbial communities lead to either divergent or convergent patterns in the decomposition process [6,7,8,9].

UV radiation has been demonstrated to accelerate the release of organic C during litter decomposition [10,11,12,13]. Photochemical reactions produce volatile compounds through photo-oxidation, which enhances the biodegradability of litter and aids subsequent microbial decomposition [1,12,14,15]. Consequently, photo-oxidation promotes the overall decay rates in litter decomposition [13,16,17,18,19]. Recently, photodegradation has been recognized as a significant driver of litter decomposition in relatively humid forests [20,21,22,23]. However, the mechanisms underlying the combined effects of UV radiation, litter chemistry, and microbial communities on the chemical complexity of litter remain unclear.

The initial chemical composition of litter links plants’ functional traits with the decomposability of litter, playing a key role in the decomposition process [24,25]. The initial litter chemistry (C:N ratio, lignin:N ratio, and lignin content) influences the turnover rate of organic C during decomposition [26,27,28]. The initial litter chemistry hypothesis posits that the initial chemical composition continuously affects its chemical complexity during decomposition [7,9]. These initial chemical differences persist throughout decomposition, regardless of the decomposer community structure or environmental conditions, due to the association with plant phylogeny [7]. The chemical divergence hypothesis posits that the chemical composition of litter diverges during decomposition, driven by persistent initial chemical differences [8,9,29].

Some studies focus on the effects of interspecific variations in plants’ chemical traits on litter decomposition. However, these studies often overlook the impact of the initial litter chemistry variations caused by UV radiation stress on decomposition. Physiological adaptations to UV radiation induce chemical changes in plants that alter the initial chemical composition of plant litter before decomposition. To protect themselves from UV radiation damage, plants accumulate secondary metabolites, such as flavonoids and sinapate esters [30]. A meta-analysis of the physiological and growth responses of plants on the Tibetan Plateau to UV-B radiation found that with increased exposure to UV-B, the content of UV-B-absorbing compounds also increased [31].

Traditional decomposition models, which focus on microbial metabolism, propose that labile organic C degrades early, while recalcitrant organic C accumulates later during decomposition [3]. These models support the chemical convergence hypothesis, which posits that differences in litter chemistry diminish over time, eventually converging to a common chemical composition [32,33]. Despite the potential taxonomic differences among soil microbial communities, decomposers share a restricted set of biochemical pathways, which constrains the complexity of litter’s chemical composition [7,32].

Litter decomposition is influenced not only by the initial litter chemistry and decay time but also by the functional traits of the decomposer communities. Microbial life-history traits such as the growth rate, resource acquisition, and stress tolerance, as well as community assembly processes like competition, facilitation, and dispersal, play significant roles in litter decomposition [34,35]. The decomposer control hypothesis suggests that distinct decomposer communities act as functionally distinct decomposition sieves, leading to persistent variations in litter’s chemical composition [7,36]. The taxonomic diversity and metabolic complexity of microorganisms can be simplified through life-history categorization, with copiotrophs growing faster and decomposing labile organic C, while oligotrophs utilize resources efficiently at the cost of slower growth rates, decomposing recalcitrant organic C [37,38,39]. Thus, the life-history strategies of microorganisms, characterized by their substrate utilization strategies and growth rates, result in differing capabilities in terms of decomposing organic C [36,37].

The ozone layer over the Qinghai–Tibetan Plateau (QTP) typically exhibits an ozone valley from May to September, with total column ozone (TCO) values approximately 10% lower than those in other regions at similar latitudes [40]. The daily average UV radiation levels on the plateau are higher compared to those at similar latitudes elsewhere [41]. Furthermore, stratospheric ozone regulates the global radiation balance by absorbing shortwave radiation and both absorbing and emitting longwave radiation [40]. Human-induced changes in the ozone layer increase the UV-B radiation dose received by organisms in high-altitude areas [42].

UV radiation not only facilitates the breakdown of litter organic C through photochemical mineralization but also influences its decomposition by interacting with soil microorganisms and litter chemistry. This research aims to investigate how the initial chemical components of plant litter, microbial communities, and UV radiation interact to affect the chemical complexity and decomposition of organic C in litter. Specifically, this study seeks to (1) identify patterns of divergence or convergence in litter’s chemical complexity during decomposition; (2) examine the effects of the initial litter chemistry, microbial communities, and UV radiation on chemical complexity during decomposition; and (3) explore the abiotic and biotic factors that impact the chemical complexity, thereby clarifying the chemical and microbial mechanisms that drive these patterns.

## 2. Materials and Methods

### 2.1. Study Site

This study was conducted in the eastern Qinghai–Tibetan Plateau (QTP), specifically in Hongyuan County, Sichuan Province, China, at an altitude of 3500 m (32°48′ N, 102°33′ E). This alpine ecosystem features short, cool summers and long, cold winters. The mean annual temperature is 1.1 °C, with the maximum average monthly temperature being 10.9 °C (July), and the minimum average monthly temperature is −10.3 °C (January). The mean annual precipitation is 753 mm, primarily occurring from May to September [43]. The peak UV radiation intensity at noon during summer reaches 3564 μW/cm^2^ and the photosynthetically active radiation (PAR) intensity can be as high as 50,700 μW/cm^2^. The soil at the study site is classified as Histosols [44], has a pH ranging from 6.6 to 7.0, and contains total carbon and total nitrogen concentrations ranging from 370 to 450 g kg^−1^ and from 13 to 19 g kg^−1^, respectively, in the top 0–10 cm of the soil [45]. The major vegetation type is alpine marsh meadow. The vegetative cover exceeds 90%, primarily comprising sedge species such as *Kobresia tibetica* (KT), *Kobresia humilis*, *Kobresia pygmaea*, *Carex muliensis*, *Scirpus pumilus*, and *Blysmus sinocompressus*, complemented by grasses like *Deschampsia caespitosa* (DC)*, Poa pratensis*, and *Agrostis matsumurae*, and forbs including *Chamaesium paradoxum* and *Anemone trullifolia* var. linearis [46].

### 2.2. Experimental Design

This study aimed to assess the effect of UV radiation on plants’ chemical composition throughout the growth phase and to explore changes in the litter chemistry due to the combined interactions between UV radiation and soil microorganisms during decomposition. To this end, we established a UV attenuation experiment in a *K. tibetica*-dominated ecosystem, constructing ten pairs of frames (150 cm × 80 cm × 35 cm; L × W × H) equipped with plastic louvers that either transmitted (KTPass) or blocked (KTBlock) UV radiation (Figure S1a) [13,47]. These louvers employed two types of filters: a UV-transmitting type (transparent acrylic) that allowed 90% of UV and 95% of PAR radiation to pass through, and a UV-blocking type (UV-absorbing polycarbonate) that blocked 90% of UV radiation while transmitting 95% of PAR. A UV radiometer equipped with UV-B and UV-A probes was used to evaluate the spectral emissions of both UV-B and UV-A radiation, and a radiometer equipped with a PAR probe was also used to assess the spectral output of PAR radiation (Photoelectric Instrument Factory of Beijing Normal University, Beijing, China). The experimental setup was randomly positioned over the herbaceous vegetation throughout its growth phase.

We collected litter from *K. tibetica* exposed to UV-transmitting and UV-blocking conditions (KTPass and KTBlock), maintaining a 10 cm buffer around our experimental setup to minimize the edge effects. Additionally, litter from *K. tibetica* under ambient solar radiation outside the experimental setup served as a control (KTCK), along with litter from *D. caespitosa*, a subdominant herb with distinct litter chemistry (DCCK). In summary, we gathered litter representing four distinct litter chemistries before decomposition (Figure S1b): *K. tibetica* subjected to UV-pass and UV-block conditions (KTPass and KTBlock), and *D. caespitosa* and *K. tibetica* both grown under ambient solar radiation (DCCK and KTCK). These materials were selected based on their significant contribution to ecosystem biomass and heterogeneous litter chemistries. All the litter was harvested 10 cm above the soil to prevent interaction with soil organisms, then air-dried and stored in darkness at room temperature.

Following autumn senescence, litter falling to the ground can be covered by subsequently fallen plant litter, referred to here as bottom litter. To simulate the combined effects of UV radiation and soil microorganisms on the chemical composition of bottom litter during decomposition, we placed 8 g of each initial litter chemistry into 2 mm mesh bags (20 cm × 20 cm) and secured them to the ground with iron stakes, covered by litter layers (Figure S1c). UV-transmitting and UV-blocking treatments were applied using the previously described attenuation devices, resulting in an array of 120 litter bags (4 initial litter chemistries × 2 UV treatments × 3 sampling times × 5 replicates).

To verify the effectiveness of the UV attenuation devices, we systematically monitored the radiation levels beneath them and regularly trimmed the vegetation to prevent interference with solar radiation by plants growing in the following year. The soil samples and litter bags were collected in May, August, and November of the next year. After collection, we removed soil clods and plant debris from the litter bags. Subsequently, we quantified the litter decomposition by measuring the mass loss, characterized the chemical composition of organic C, and analyzed the soil microbial community composition and extracellular enzyme activity.

### 2.3. Solid-State ^13^C Cross-Polarization Magic-Angle Spinning Nuclear Magnetic Resonance Spectroscopy

In this study, we utilized solid-state ^13^C cross-polarization magic-angle spinning nuclear magnetic resonance (^13^C-CPMAS NMR) to characterize plant litter under consistent experimental conditions. Litter samples were analyzed at four different recycling times across various decomposition stages (0, 222, 314, and 397 days). We employed a Bruker Avance III 400 spectrometer (Bruker BioSpin, Fällanden, Switzerland), operating at a frequency of 100.6 MHz for our measurements. The samples were contained within a 4 mm cylindrical zirconium rotor with Kel-F end-caps and examined using a double resonance probe. The NMR spectra were recorded at a spinning frequency of 10 kHz, recorded with a recycle time of 1 s, an acquisition time of 20 ms, and 2000 scans. To circumvent the non-homogeneity of the Hartmann–Hahn condition at high rates of the spin rotor, a ^1^H ramp sequence was applied before transference to the ^13^C using a 1 ms contact time. Moreover, we calibrated the C chemical shift using the methylene signal of solid adamantane at 29.5 ppm as an external standard [48,49]. This approach ensured the accurate and reliable characterization of the litter’s chemical composition, essential for understanding litter decomposition.

The NMR spectra were integrated to calculate the peak areas within the designated chemical shift regions corresponding to specific C types. Seven chemical shift regions were identified corresponding to the following C types [50]: alkyl C (0–45 ppm), N-alkyl and methoxyl C (46–60 ppm), O-alkyl C (61–90 ppm), di-O-alkyl C (91–110 ppm), H,C-substituted aromatic C (111–140 ppm), O-substituted aromatic C (141–160 ppm) and carbonyl C (161–190 ppm). The detailed calculation methods are provided in the Supplementary Materials. The O-C-O (di-O-alkyl C) and O-C (O-alkyl C) bonds are characteristic of carbohydrates, indicating that both cellulose and certain hemicelluloses generate a common peak or signal [51,52]. O-substituted aromatic carbon, represented by the phenolic C-O bond, is found in guaiacyl lignin, condensed tannins, and other phenolic compounds [53,54]. This substitution typically occurs at carbon positions 3, 4, or 5 when the R group is a hydroxyl (–OH) group [53].

### 2.4. Microbial Community Characterization and Bipartite Network Analysis

To investigate the bacterial and fungal community composition during decomposition, we extracted the total genomic DNA from 1 g of soil per sample using the PowerMax Soil DNA Isolation Kit (MO BIO, Carlsbad, CA, USA). The DNA quality and concentration were assessed with a NanoDrop 2000 Spectrophotometer (Thermo Scientific, Waltham, MA, USA). For the bacterial 16S rRNA gene analysis, the V4 hypervariable region was amplified using the primer sets 515F (5′-GTGCCAGCMGCCGCGGTAA-3′) and 909R (5′-CCCCGYCAATTCMTTTRAGT-3′). For the fungal ITS rRNA analysis, the ITS4 (5′-TCCTCCGCTTATTGATATGC-3′) and gITS7F (5′-GTGARTCATCGARTCTTTG-3′) primers were employed. 

Bipartite networks were constructed using the R packages psych (version 2.2.5) and igraph (version 1.3.1) to examine the ecological interactions between the operational taxonomic units (OTUs) and litter organic C. Spearman’s correlation coefficients were used to evaluate the pairwise correlations, with significance defined at r > 0.6 and *p* < 0.05 for the network construction.

### 2.5. Extracellular Enzyme Activity Assays

Plant litter’s cell walls are primarily composed of cellulose, hemicellulose, and lignin. Hence, we measured the cellulose and hemicellulose hydrolase, and two representatives of lignin oxidases. The extracellular enzymes were categorized into three functional groups: hydrolytic enzymes for cellulose degradation (cellobiohydrolase, CBH, and β-1,4-glucosidase, BG), hydrolytic enzymes for hemicellulose degradation (β-1,4-xylosidase, BX), and oxidative enzymes for lignin degradation (phenol oxidase, PHO, and peroxidase, PERO) [6]. Additionally, we measured the nitrate reductase (NR) for nitrogen cycling, and the acid phosphatase (ACP) and alkaline phosphatase (ALP) for phosphorus cycling. The extracellular enzyme activity was expressed as μmol h^−1^ g^−1^ dry soil, with the detailed assay procedures cited in [55]. 

### 2.6. Statistical Analyses

Principal coordinate analysis (PCoA) was used to assess the complexity of the litter’s chemical composition, with four initial chemistries under two UV radiation treatments across three decomposition stages. The patterns in the litter chemistry between any two samples were measured using the Bray–Curtis distance, based on nuclear magnetic resonance (NMR) datasets, reflecting the litter’s chemical complexity during decomposition [6]. We conducted permutational multivariate analysis of variance (PERMANOVA) with the Bray–Curtis distance to assess the effects of UV radiation, initial litter chemistry, and decay time on litter’s chemical complexity. Distance-based redundancy analysis (dbRDA), followed by variation partitioning analysis (VPA), was used to determine the contributions of environmental variables to the overall variations in the litter’s chemical complexity. Mantel tests were conducted to assess the relationship between the litter’s chemical complexity and the soil enzyme activity.

The decomposition patterns were described using the exponential equation, k = −ln(X_t_/X_0_)/t, where X_t_ represents the mass remaining at time t, k is the decay constant (year^−1^), and X_0_ is the initial dry mass. A three-way analysis of variance (ANOVA) was conducted to evaluate the influence of the UV radiation, initial litter chemistry, and decay time on the litter mass loss, organic C, and extracellular enzyme activity. For normally distributed data, multiple comparisons were conducted using Tukey’s honest significant difference (HSD) test. For non-normally distributed data, the Kruskal–Wallis test was applied, followed by multiple comparisons using the Nemenyi test. The effects of UV radiation on the litter mass and organic C were calculated using the natural logarithm of the response ratio (lnR). A detailed description of the calculation is included in the Supplementary Materials. 

Linear models estimated through ordinary least squares (OLS) were used to predict the relationship between the initial relative abundance of C functional groups and the effect of UV radiation on the litter mass loss. Random forest regression was utilized to assess the relative contribution of the decay indices (organic C based on NMR datasets) to the litter mass loss, focusing on the importance of variables. The importance of variables is ranked based on the increase in node purity, which refers to the decrease in the residual sum of squares resulting from using the variable to split the regression tree. All the statistical analyses and graphical representations were conducted in the R computing environment version 4.2.0 (R Development Core Team, 2022), utilizing packages such as vegan (version 2.6-2), ape (version 5.6-2), linkET (version 0.0.7.1), microeco (version 0.13.0), metafor (version 3.4-0), PMCMRplus (version 1.9.10), randomForest (version 4.7-1.1), rfPermute (version 2.5.1), and ggplot2 (version 3.4.4). 

## 3. Results

### 3.1. Effect of UV Radiation on Plants’ Chemical Traits during the Growth Phase

**Figure 1:**
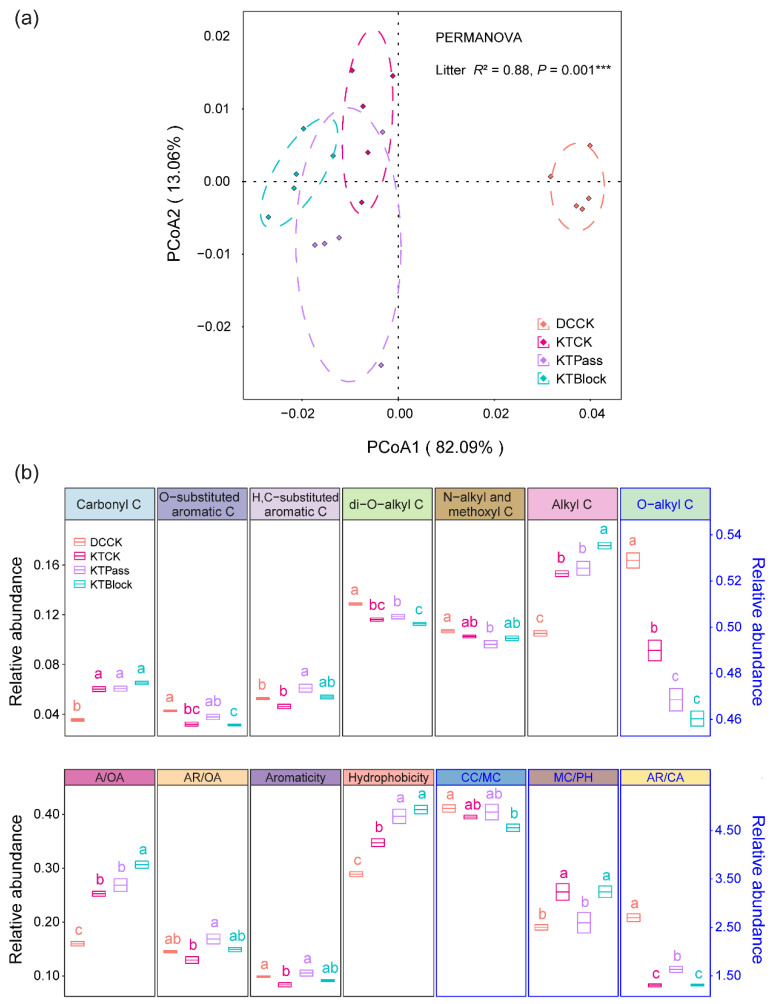
Chemical complexity (**a**) and initial relative abundance of organic carbon (**b**) before decomposition. PCoA based on the Bray–Curtis distances illustrates the complexity of the initial litter chemistry. The dot colors denote the initial litter chemistries, with dotted ellipse borders indicating 65% confidence intervals. Permutational multivariate analysis of variance (PERMANOVA) with the Bray–Curtis distance to assess the effect of initial litter chemistry on the chemical complexity (*** *p* = 0.001). The litter materials were classified into four distinct initial chemistries before decomposition: *Deschampsia caespitosa* (DCCK) and *Kobresia tibetica* (KTCK), both grown under ambient solar radiation (CK), and *K. tibetica* (KTPass and KTBlock), subjected to UV-pass and UV-block treatments, respectively. The letters indicate significant differences among the initial litter chemistries (mean ± SE, *n* = 5, Tukey’s HSD, *p* < 0.05). The organic carbon and decay indices in the black frames correspond to the left axis, while those in the blue frames correspond to the right axis.

The initial litter chemistries significantly influenced the chemical complexity of the litter organic C, showing divergent patterns before decomposition (R^2^ = 0.88, *p* = 0.001, Figure 1a). Significant differences were observed in the chemical complexity of the litter organic C across the distinct initial litter chemistries (Table S1). Additionally, the initial relative abundances of O-substituted aromatic C, di-O-alkyl C, O-alkyl C, AR/CA, and Aromaticity in *D. caespitosa* were higher than those in *K. tibetica* after growth under ambient solar radiation (*p* < 0.05, Figure 1b). Conversely, the initial relative abundances of carbonyl C, alkyl C, A/OA, and MC/PH, and the hydrophobicity, in *D. caespitosa* were lower than those in *K. tibetica* (*p* < 0.05, Figure 1b). In comparison, under the UV radiation attenuation treatments, UV radiation increased the initial relative abundances of O-substituted aromatic C, di-O-alkyl C, and AR/CA, while decreasing those of alkyl C, A/OA, and MC/PH in *K. tibetica* (*p* < 0.05, Figure 1b). 

### 3.2. Effect of UV Radiation on Litter’s Chemical Traits during the Decomposition Phase

**Figure 2:**
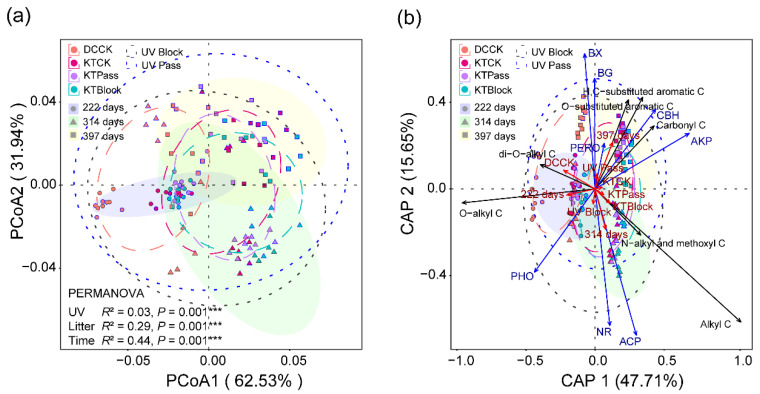
Changes in litter’s chemical complexity during decomposition. PCoA (**a**) and dbRDA (**b**) dissimilarity matrices based on the Bray–Curtis distances reveal divergent patterns in the litter’s chemical complexity, influenced by the UV radiation, initial litter chemistries, and decay time. Permutational multivariate analysis of variance (PERMANOVA) with the Bray–Curtis distance to assess the effects of UV radiation, initial litter chemistry, and decay time on the chemical complexity (*** *p* = 0.001). The litter materials were classified into four distinct initial chemistries before decomposition: *Deschampsia caespitosa* (DCCK) and *Kobresia tibetica* (KTCK), both grown under ambient solar radiation (CK), and *K. tibetica* (KTPass and KTBlock), subjected to UV-pass and UV-block treatments, respectively. The UV radiation, initial litter chemistries, and decay time are represented as UV, Litter, and Time. The decomposition stages at 222, 314, and 397 days represent the early, mid, and late stages, symbolized by circles, triangles, and squares. The dot fills represent the initial litter chemistries, and the dot colors denote the UV radiation treatments. The shaded ellipses, blue and black dotted ellipse borders, represent 95% confidence intervals, with the other dotted ellipse borders indicating 65% confidence intervals.

Throughout the decomposition continuum, the chemical composition of the litter organic C showed divergent patterns (Figure 2a,b). The UV radiation, initial litter chemistry, and decay time significantly influenced the complexity of the litter chemistry (Figure 2a, Table S2). There were significant differences in the litter’s chemical complexity between DCCK, KTCK, KTPass, and KTBlock (Table S2). Although the initial litter chemistry and decay time did not alter the effect of UV radiation on the relative abundance of the litter organic C (Figure S2b,c), the functional groups of organic C significantly influenced the magnitude of the effect size of UV radiation on the relative abundance of organic C (*p* = 0.0042, Figure S2a). UV radiation increased the relative abundances of carbonyl C, O-substituted aromatic C, and H, C-substituted aromatic C (Figure S2a). However, UV radiation had no effect on the relative abundance of di-O-alkyl C, O-alkyl C, N-alkyl and methoxyl C, and alkyl C (Figure S2a).

The relative abundances of di-O-alkyl C and O-alkyl C from DCCK were higher than those in KTCK across all the stages of decomposition, regardless of the UV radiation exposure (*p* < 0.05, Figure S3, Table S5). Conversely, the relative abundances of carbonyl C and alkyl C were lower in DCCK compared to KTCK across all the stages of decomposition (*p* < 0.05, Figure S3). However, there was no significant difference in these C types between KTPass and KTBlock (Figure S3). Other C types, including O-substituted aromatic C, H, C-substituted aromatic C, and N-alkyl and methoxyl C, displayed no significant difference in relative abundances between DCCK and KTCK, nor between KTPass and KTBlock, at any stage of decomposition (Figure S3). 

### 3.3. Relationships between Initial Litter Chemistry and Decay Rate

**Figure 3:**
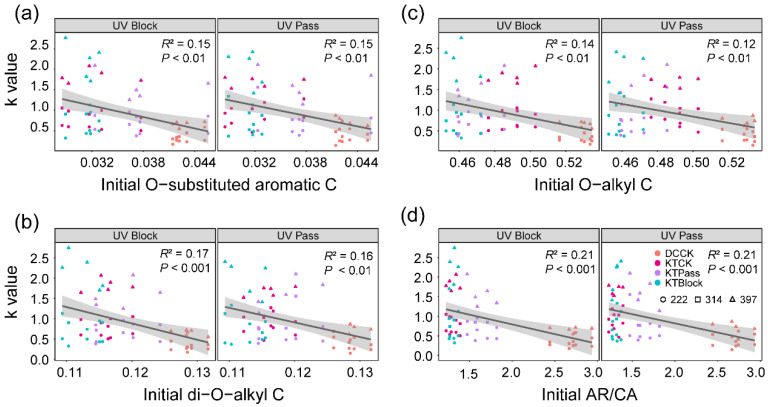
Relationships between the k value and the initial litter chemical composition during decomposition. The initial relative abundances of O-substituted aromatic C (**a**), di-O-alkyl C (**b**), and O-alkyl C (**c**), along with the initial ratio of aromatic C to carbonyl C (**d**) correlated with k values during litter decomposition. The litter materials were classified into four distinct initial chemistries before decomposition: *Deschampsia caespitosa* (DCCK) and *Kobresia tibetica* (KTCK), both grown under ambient solar radiation (CK), and *K. tibetica* (KTPass and KTBlock), subjected to UV-pass and UV-block treatments, respectively. The solid lines denote significant relationships (*p* < 0.05), with shading indicating 95% confidence bands of the fitted lines. The point colors represent the initial litter chemistries, and the shapes denote the different decomposition stages at 222, 314, and 397 days (early, mid, and late stages) (**a**–**d**).

The initial relative abundance of O-substituted aromatic C exhibited negative correlations with the decay rate constant (k value) under both UV-block (*R*^2^ = 0.15, *p* < 0.01, Figure 3a) and UV-pass conditions (*R*^2^ = 0.15, *p* < 0.01, Figure 3a). Similarly, negative associations were observed between the initial relative abundance of di-O-alkyl C and the k value under both UV-block (*R*^2^ = 0.17, *p* < 0.001, Figure 3b) and UV-pass conditions (*R*^2^ = 0.16, *p* < 0.01, Figure 3b). Additionally, the initial relative abundance of O-alkyl C showed negative correlations with the k value under both UV-block (*R*^2^ = 0.14, *p* < 0.01, Figure 3c) and UV-pass conditions (*R*^2^ = 0.12, *p* < 0.01, Figure 3c). Furthermore, the initial ratio of aromatic C to carbonyl C (AR/CA) negatively correlated with the k value under both UV-block (*R*^2^ = 0.21, *p* < 0.001, Figure 3d) and UV-pass conditions (*R*^2^ = 0.21, *p* < 0.001, Figure 3d). However, no significant relationship was found between the initial abundance of the litter’s chemical composition and the effect of UV radiation on the litter mass loss (Table S3).

### 3.4. Relative Contribution of Litter’s Chemical Components as Predictors of Mass Loss

UV radiation did not significantly affect the mass loss across the distinct initial litter chemistries throughout the decomposition process (Figure S4, Table S6). Over 397 days of litter decomposition, the mass loss percentages of DCCK, KTCK, KTPass, and KTBlock exposed to UV-pass conditions were 55%, 84%, 82%, and 88%, respectively (Figure S5). Under UV-block conditions, the mass loss percentages were 52%, 86%, 81%, and 90% for DCCK, KTCK, KTPass, and KTBlock, respectively (Figure S5). Throughout the decomposition stages, KTCK consistently showed higher mass loss compared to DCCK, regardless of the UV radiation exposure (*p* < 0.05, Figure S5). There was no significant difference in the mass loss between KTPass and KTBlock under UV-pass conditions at any decomposition stage (Figure S5). However, KTPass exhibited lower mass loss than KTBlock under UV-block conditions at 397 days of decomposition (*p* < 0.05, Figure S5). 

**Figure 4:**
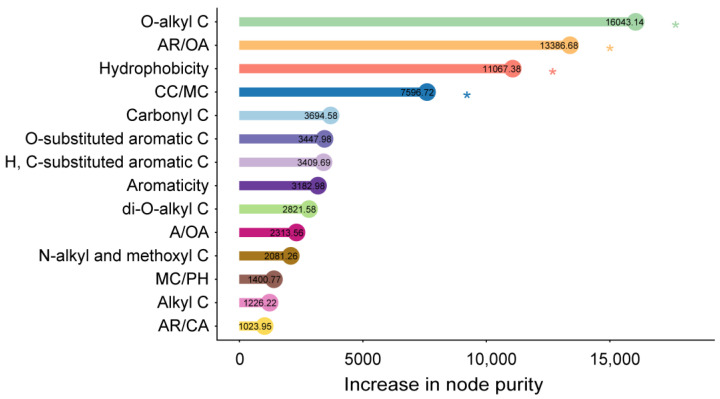
Relative contribution of the decay indices based on the chemical composition to the litter mass loss. Random forest regression analysis was conducted on the organic carbon abundances from decomposing litter. The importance of variables was assessed by the increase in node purity, with higher values indicating greater importance (* *p* < 0.05).

The regression models accounted for 86% of the variation in the mass loss during litter decomposition (Figure 4). The primary decay indices identified by random forest regression as predictors of mass loss included the O-alkyl C, AR/OA, hydrophobicity, and CC/MC in the litter materials (Figure 4). Furthermore, the different litter chemical components showed varied trends throughout the decomposition process. The relative abundances of carbonyl C, O-substituted aromatic C, and H, C-substituted aromatic C increased over time (Figure S6a–c). Conversely, the relative abundances of di-O-alkyl C and O-alkyl C decreased over time (Figure S6d,e). The decay indices related to litter decomposability, such as A/OA, AR/OA, AR/CA, aromaticity, and hydrophobicity, were positively correlated with the decay time (Figure S6h, k–n). Conversely, CC/MC and CC/PH were negatively associated with the decay time (Figure S6i,j).

### 3.5. Relationships between Extracellular Enzyme Activity and Litter’s Chemical Complexity

Exposure to UV radiation decreased the activities of several extracellular enzymes during litter decomposition. Specifically, the activities of phenol oxidase (PHO) and β-1,4-glucosidase (BG) decreased at 314 days. Nitrate reductase (NR) activity decreased at both 314 and 397 days, and acid phosphatase (ACP) activity decreased at 222, 314, and 397 days. Furthermore, cellobiohydrolase (CBH) activity increased over time (*p* < 0.05, Figure S7). The activities of β-1,4-xylosidase (BX) and β-1,4-glucosidase (BG) declined at 314 days but increased at 397 days (*p* < 0.05, Figure S7). Phenol oxidase (PHO) activity remained constant at both 222 and 314 days, with a reduction observed at 397 days (*p* < 0.05, Figure S7). 

**Figure 5:**
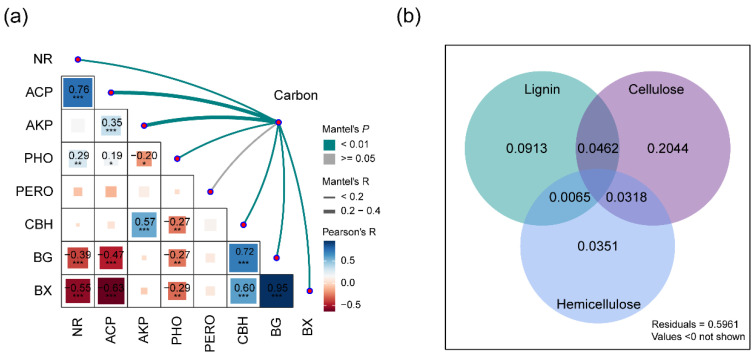
Mantel tests and variance partitioning analysis (VPA) of the litter’s chemical complexity and soil enzyme activities. (**a**) Mantel tests show relationships, with the edge width proportional to the Mantel’s R value and the edge color indicating statistical significance. Pairwise correlations are depicted with a color gradient representing Pearson’s correlation coefficients. The numbers represent significant Pearson correlation coefficients. The enzyme activities include cellobiohydrolase (CBH), β-1,4-glucosidase (BG), β-1,4-xylosidase (BX), phenol oxidase (PHO), peroxidase (PERO), acid phosphatase (ACP), alkaline phosphatase (ALP), and nitrate reductase (NR). (**b**) VPA illustrates the proportion of the variation in the litter’s chemical complexity explained by environmental variables. The shades represent the combined effects of environmental variables on the variation in the litter’s chemical complexity. The extracellular enzyme activities are categorized by their role in degrading lignin (PHO, PERO), cellulose (CBH, BG), and hemicellulose (BX). Significance levels are as follows: *** 0.001, ** 0.01, * 0.05.

The litter’s chemical complexity increased with changes in the extracellular enzyme activity (R = 0.34, *p* = 0.001). Positive correlations were observed between the litter’s chemical complexity and the activities of several extracellular enzymes, including phenol oxidase (PHO), cellobiohydrolase (CBH), β-1,4-glucosidase (BG), β-1,4-xylosidase (BX), acid phosphatase (ACP), alkaline phosphatase (ALP), and nitrate reductase (NR), with the exception of peroxidase (PERO) (Figure 5a, Table S4). Additionally, the activities of enzymes involved in the degradation of lignin, cellulose, and hemicellulose accounted for 9%, 20%, and 4% of the variance in the litter’s chemical complexity throughout the decomposition process, respectively (Figure 5b).

### 3.6. Changes in Microbial Community Composition and Bipartite Networks

The bacterial community was primarily composed of Acidobacteria, with relative abundances of 33%, 37%, and 31% at 222, 314, and 397 days of decomposition, respectively, followed by Proteobacteria with 31%, 29%, and 26% (Figure S8a). In contrast, the fungal community was dominated by Ascomycota, accounting for 57%, 61%, and 75% at 222, 314, and 397 days, respectively, followed by Basidiomycota at 23%, 18%, and 19% (Figure S8b). UV radiation significantly decreased the relative abundance of Acidobacteria at 314 days (*p* < 0.05, Figure S9a), whereas it significantly increased the relative abundance of Ascomycota at this stage (*p* < 0.05, Figure S9b). Moreover, the relative abundance of Acidobacteria increased at 314 days but decreased at 397 days (*p* < 0.05, Figure S9a). The relative abundance of Planctomycetes increased over time (*p* < 0.05, Figure S9a). Conversely, the relative abundance of Actinobacteria decreased over time (*p* < 0.05, Figure S9a).

**Figure 6:**
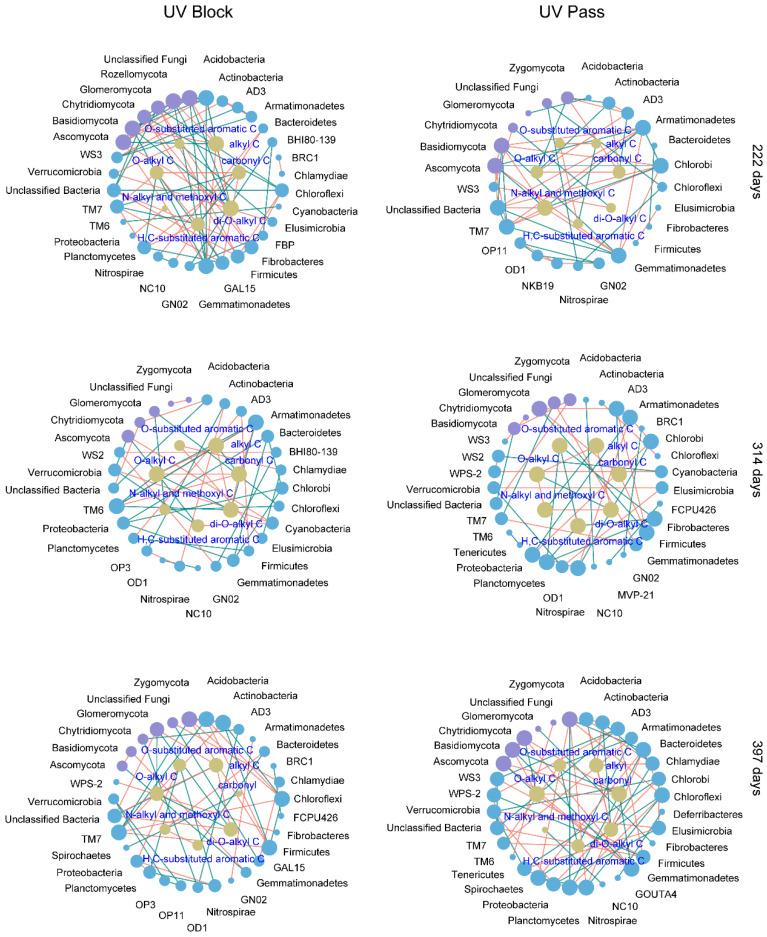
Bipartite networks between the litter’s organic carbon (C) and the soil microorganisms in the UV radiation attenuation treatments during decomposition. The bode colors indicate taxonomic groups at the phylum level (blue and purple nodes for bacteria and fungi phyla) and litter organic C (yellowish–brown nodes for ^13^C NMR-based C types). The node sizes reflect the node degree, with larger nodes indicating higher degrees. The line colors represent significant positive (green) and negative (red) relationships between the soil microorganisms and the litter’s chemistry.

Over a 222-day stage of litter decomposition under UV-block conditions, O-alkyl C exhibited a positive association with GAL15 and Planctomycetes. Di-O-alkyl was correlated positively with Acidobacteria, Gemmatimonadetes, Basidiomycota, and Rozellomycota. Carbonyl C and H, C-substituted aromatic C were positively associated with TM7. O-substituted aromatic C was positively correlated with Elusimicrobia. Alkyl C was positively associated with Ascomycota. Under UV-pass conditions, O-alkyl C was positively correlated with Actinobacteria and Zygomycota. Di-O-alkyl C was positively associated with Chytridiomycota. Carbonyl C was positively correlated with AD3. N-alkyl and methoxyl C was positively associated with Armatimonadetes, Chlorobi, and Ascomycota (Figure 6).

At 314 days of decomposition, under UV-block conditions, positive associations emerged between di-O-alkyl C and O-alkyl C with both Armatimonadetes and TM6. H, C-substituted aromatic C was positively associated with Verrucomicrobia and Ascomycota. N-alkyl and methoxyl C were positively correlated with Chloroflexi and Glomeromycota. Under UV-pass conditions, positive correlations emerged between O-substituted aromatic C and H, C-substituted aromatic C with both Fibrobacteres and Planctomycetes. Carbonyl C was positively associated with AD3 (Figure 6).

After 397 days of decomposition, under UV-block conditions, O-alkyl C and di-O-alkyl C had positive relationships with Glomeromycota. O-substituted aromatic C was positively associated with Nitrospirae and Planctomycota. H, C-substituted aromatic C was positively correlated with Bacteroidetes. Carbonyl C was positively related to Spirochaetes. Under UV-pass conditions, di-O-alkyl C showed positive associations with Actinobacteria and Chlamydiae. O-alkyl C was positively associated with Ascomycota and Chytridiomycota. Both O-substituted aromatic C and H, C-substituted aromatic C had positive correlations with Armatimonadetes. Carbonyl C was positively associated with Gemmatimonadetes and Basidiomycota. Alkyl C was positively correlated with Acidobacteria (Figure 6). 

## 4. Discussion

### 4.1. Initial Chemical Traits Influence Litter Decomposition

Litter, composed of diverse organic carbon (C) compounds, poses significant challenges in terms of quantifying its chemical components due to its complexity [7,56]. ^13^C NMR is used to evaluate the structure and abundance of organic C in plant litter, to explore the mechanisms of abiotic and biotic factors in litter decomposition at the chemical molecular level, and to overcome the limitations of studying potential decomposition mechanisms by analyzing the loss of litter mass, lignin, cellulose, and hemicellulose. Our study found that the chemical composition of litter organic C diverged throughout the decomposition, influenced by the initial litter chemistry and UV radiation. Notably, *D. caespitosa* consistently exhibited higher relative abundances of di-O-alkyl C and O-alkyl C compared to *K. tibetica* at all the decomposition stages, regardless of the UV exposure. These findings support the initial chemistry and divergence hypothesis, suggesting that differences in the initial chemical composition of litter persist throughout decomposition, leading to divergence in the chemical complexity during decomposition. This concept aligns with previous studies highlighting the role of the initial chemical differences in fostering chemical divergence during decomposition [7,8,9,29]. Additionally, some studies have found that the initial recalcitrant compounds contribute to the chemical divergence in litter [7,9]. However, our findings indicate that differences in labile compounds, specifically di-O-alkyl C and O-alkyl C, led to this divergence.

The degradability of organic C compounds in litter significantly influences the decay rates [48,57]. The decay rates correlate with the initial C:N, lignin:N, or lignin:cellulose ratios [26,27,28,58], explaining 73% of global decay rate variations [59]. Specifically, the decay rates are inversely correlated with the initial lignin content or lignin:cellulose ratios [28,59]. Our findings corroborate this correlation at the molecular level, revealing a negative relationship between the decay constant (k value) and the initial relative abundance of O-substituted aromatic C in lignin. Moreover, we find that the k values negatively associate with the initial relative abundances of di-O-alkyl C and O-alkyl C, and the initial aromatic C to carbonyl C ratio.

### 4.2. Decay Time and Its Influence on Litter Decomposition

The chemical convergence hypothesis posits that the litter chemistry will ultimately align with a uniform set of recalcitrant characteristics over time, irrespective of the initial chemical variations [32,33]. Contrarily, our results challenge this hypothesis, displaying divergent chemical compositions during a one-year decomposition. Our findings are consistent with previous research indicating that the chemical composition of litter organic C diverges during a one-year decomposition period [9,29]. Additionally, recent research indicates that litter’s chemical complexity evolves dynamically, showing divergence in the short term (up to three years) and convergence in the long term (beyond nine years) [6]. 

Lignin acts as a protective barrier for cellulose and hemicellulose in the plant cell wall, enhancing resistance to microbial decay [28]. Guaiacyl units in lignin form condensed (5,5′) aryl–aryl linkages, while syringyl-type lignin contains more labile β-O-4 linkages [56,57]. Aryl–aryl linkages tend to persist through soil formation, whereas other linkage types decompose rapidly during the initial stages of litter decomposition [57]. Our one-year observations showed that a decline in di-O-alkyl C and O-alkyl C, and an increase in O-substituted aromatic C and H, C-substituted aromatic C, with an increased decay time. This pattern aligns with the traditional litter decomposition model, where labile organic C degrades swiftly in the early stages, leading to the loss of soluble compounds through leaching; in contrast, in the later stage of decomposition, recalcitrant organic C gradually accumulates, limiting the decay rates [49,60].

### 4.3. Microbial Communities and UV Radiation Influence Litter Decomposition

UV radiation did not significantly affect the mass loss or the relative abundances of specific C types in the bottom litter during decomposition, but it increased the relative abundances of carbonyl C, O-substituted aromatic C, and H, C-substituted aromatic C. These findings suggest that UV photodegradation has minimal effects on litter decomposition at the mass levels, likely due to the varying intensities of photodegradation related to the litter layer thickness [16]. Furthermore, UV radiation increased the relative abundance of aromatic C because it inhibited the activity of phenol oxidase related to lignin decomposition. The main contributor to the litter mass loss is the decomposition of labile O-alkyl C, while recalcitrant aromatic C accumulates, confirming established decomposition models [3,53]. Moreover, the ratios of alkyl C to O-alkyl C (A/OA) and carbohydrates to methoxy C (CC/MC) serve as reliable predictors of the decomposition rates when utilizing solid-state NMR techniques [48,61,62].

The increased availability of specific C resources enhances the activity of decomposers specialized in breaking down substances like cellulose, hemicellulose, and lignin [36]. Our findings indicated that shifts in the enzyme activities correspond to variations in the litter’s chemical complexity. Enzymes involved in lignin, cellulose, and hemicellulose degradation contributed 9%, 20%, and 4%, respectively, to the variation in the litter’s chemical complexity during decomposition. This supports the decomposer control hypothesis, which suggests that distinct decomposer communities act as functional funnels, shaping litter’s chemical composition throughout decomposition [7]. Moreover, microbial catabolism theory posits that extracellular enzyme activity influences chemical complexity, particularly driven by keystone taxa within Proteobacteria [6]. In contrast, microbial anabolism promotes chemical convergence as unique inputs assimilate into microbial biomass during decomposition [63]. A recent study supports this dual effect, where short-term decomposition displays divergent patterns due to catabolism, while long-term decomposition shows convergent patterns attributed to microbial anabolism [6]. 

Specific litter substrates are utilized by distinct decomposer functional guilds under different UV conditions. For instance, during early decomposition, Planctomycetes, Acidobacteria, Gemmatimonadetes, Basidiomycota, and Rozellomycota positively correlate with O-alkyl C and di-O-alkyl C in the UV-block treatments. Conversely, Actinobacteria, Zygomycota, and Chytridiomycota thrive in the UV-pass treatments. Copiotrophic r-strategists, characterized by rapid growth and utilization of labile C, thrive in frequently disturbed environments, including Proteobacteria, Bacteroidetes, Firmicutes, and Ascomycota [38,64]. Oligotrophic K-strategists, which grow slowly and excel in relatively stable environments [39], as seen in Acidobacteria, Planctomycetes, Chloroflexi, and Basidiomycota [38,39]. Labile C is utilized by K-strategists under UV-block conditions and by r-strategists under UV exposure during early decomposition. Hence, our findings indicate that r-strategists, characterized by rapid microbial growth and a swift response to labile C inputs, are better adapted to UV stress.

Actinobacteria, especially the genera *Streptomyces* and *Mycobacterium*, possess a comprehensive suite of genes encoding carbohydrate-active enzymes [65]. These microorganisms are adept at breaking down plant cell walls by employing enzymes from the GH1, GH2, GH3, GH5, GH6, and GH16 families, which facilitate the hydrolysis of hemicellulose, cellulose, and lignin [65,66,67]. Actinobacteria are regarded as copiotrophic r-strategists in global grasslands [68] and metabolize O-alkyl C under UV radiation, peaking in relative abundance during the early stages of decomposition.

## 5. Conclusions

A complex feedback network exists among the litter chemistry, microbial communities, and UV radiation during litter decomposition. Plant litter serves as a carbon source for microorganisms, influencing the composition and structure of the microbial community. Our results highlighted the crucial role of variations in the initial O-alkyl C, which consistently determined the litter decay rates throughout decomposition. The reduction in O-alkyl C with an increased decay time is the primary predictor of the variation in the mass loss of bottom litter, emphasizing its importance in decomposition. Our results indicated that the mass loss of bottom litter is primarily caused by microbial utilization of O-alkyl carbon. The photodegradation effect of UV on organic carbon is minimal, and UV radiation even inhibits the decomposition of aromatic carbon by soil microorganisms involved in secreting phenol oxidase. However, our study observed divergent patterns in the litter’s chemical complexity across different initial chemistries and UV radiation treatments during various decomposition stages. These inherent differences in the initial chemistries contributed to the varied chemical composition observed. UV radiation altered the microbial community composition, shifting from oligotrophic K-strategists to copiotrophic r-strategists, thereby influencing the litter’s chemical complexity. These findings elucidate how changes in the initial litter chemistry and UV radiation influence the turnover rates and chemical complexity of plant-derived C, subsequently affecting the soil microbial community structure and the stability of the soil organic C. Thus, our research contributes to a deeper understanding of the ecological drivers governing C cycling and the persistence of organic C in soils.

## Data Availability

The original contributions presented in the study are included in the article/Supplementary Material, further inquiries can be directed to the corresponding author.

## Supplementary Materials

The following supporting information can be downloaded at https://www.mdpi.com/article/10.3390/microorganisms12081535/s1, Figure S1: Litter decomposition in Zoige Alpine Meadow of the Qinghai-Tibetan Plateau; Figure S2: The effect of UV radiation on the relative abundance of litter organic carbon; Figure S3: The relative abundance of chemical components of plant litter characterized by solid-state ^13^C-CPMAS NMR spectra during decomposition; Figure S4: The effect of UV radiation on mass loss during litter decomposition; Figure S5: The mass loss of plant litter during decomposition; Figure S6: Relationships between litter chemical components and decay time during decomposition; Figure S7: Extracellular enzyme activities in soil were measured after litter decomposition under UV block and UV pass treatments for 222, 314, and 397 days; Figure S8: Microbial community composition of bacteria (a) and fungi (b) within the top 10 phyla across three stages of litter decomposition; Figure S9 Relative abundance of bacteria (a) and fungi (b) during decomposition; Table S1: Significance test of litter chemical complexity across initial chemistries of organic C during the growth phase; Table S2: Significance test of litter chemical complexity across UV radiation treatments, initial litter chemistries, and decay time during the decomposition process; Table S3: The relationship between the initial chemical components of litter organic C and lnR (mass loss), and lnR (k), respectively; Table S4: Relationships between litter chemical complexity and extracellular enzyme activities of soil microorganisms during the decomposition process; Table S5: Litter chemical composition based on solid-state ^13^C CPMAS NMR analyzed by three-way analysis of variance (ANOVA); Table S6: Mass loss of plant litter analyzed by three-way analysis of variance (ANOVA).
