# Supplementary material for: Initial Litter Chemistry and UV Radiation Drive Chemical Divergence in Litter during Decomposition
Source: Microorganisms. 2024 Jul 27;12(8):1535. doi: 10.3390/microorganisms12081535 (PMC11356187; doi:10.3390/microorganisms12081535)
Supplement: Supplementary file 1 [file microorganisms-12-01535-s001.zip › microorganisms-3059405-supplementary.pdf]

Supporting Information

Article title: **Initial litter chemistry and UV radiation drive chemical divergence in litter during decomposition**

Bei Yao[1], Xiangshi Kong[2], Kai Tian[1], Xiaoyi Zeng[1], Wenshuo Lu[1], Lu Pang[1], Shucun Sun[1], and Xingjun Tian[1, 3*]

[1]School of Life Sciences, Nanjing University, Nanjing 210023, China

[2]Key Laboratory for Ecotourism of Hunan Province, School of Tourism, Jishou University, Jishou 416000, China

[3]Co-Innovation Center for Sustainable Forestry in Southern China, Nanjing Forestry University, Nanjing 210037, China

*Author of correspondence: Xingjun Tian

Tel: +86-13851857867

E-mail: tianxj@nju.edu.cn

ORCID:

0000-0002-0251-2582 (Xingjun Tian),

0000-0001-6434-1806 (Bei Yao)

The following Supporting Information is available for this article:

2 Materials and methods

2.3 ^{13}C cross-polarization magic angle spinning nuclear magnetic resonance spectroscopy

The relative abundance of C functional groups was determined by the proportion of the area under the peaks in these regions to the total peak area, using MestreNova software (Mestrelabs Research, Santiago de Compostela, Spain). This analysis provided specific integrated regions to derive indices associated with litter decomposability. Decay rates were linked to the dynamic ratio of labile to recalcitrant C. The ratio of carbohydrate C to N-alkyl and methoxyl C (CC/MC) and the ratio of alkyl C to O-alkyl C (A/OA) were representing the proportion of labile to recalcitrant C, respectively. The ratio of N-alkyl and methoxyl C to O-substituted aromatic C (MC/PH) served to differentiate signals related to lignin and other phenolic compounds. Hydrophobicity was assessed to contrast hydrophobic and hydrophilic functional groups. Aromaticity was calculated to establish the proportion of aromatic carbon within total organic C of leaf litter [1-4].

2.6 Statistical analyses

The effect of UV radiation on litter decomposition, specifically on litter mass loss and organic C, were quantified using the natural logarithm of the response ratio (lnR) for treatments allowing UV radiation (UV pass) versus those blocking UV radiation (UV block), as follows: $\mathrm{lnR} = \ln\left(\frac{x_p}{x_b}\right)$, with $v = \frac{s_p^2}{n_p x_p^2} + \frac{s_b^2}{n_b x_b^2}$, where x_p, s_p, and n_p are the mean, standard deviation, and sample size of variables under UV pass treatment, while x_b, s_b, and n_b are the mean, standard deviation, and sample size of variables under

UV block, respectively. Here, lnR (mass loss) > 0 suggests that an increase of litter mass loss due to UV radiation, whereas lnR (mass loss) < 0 implies a decrease. The same logic applies to the effect of UV radiation on the relative abundance of litter organic C. All statistical analyses were performed using random effects models with the grouping variables of initial litter chemistries, decay time, and C functional groups.

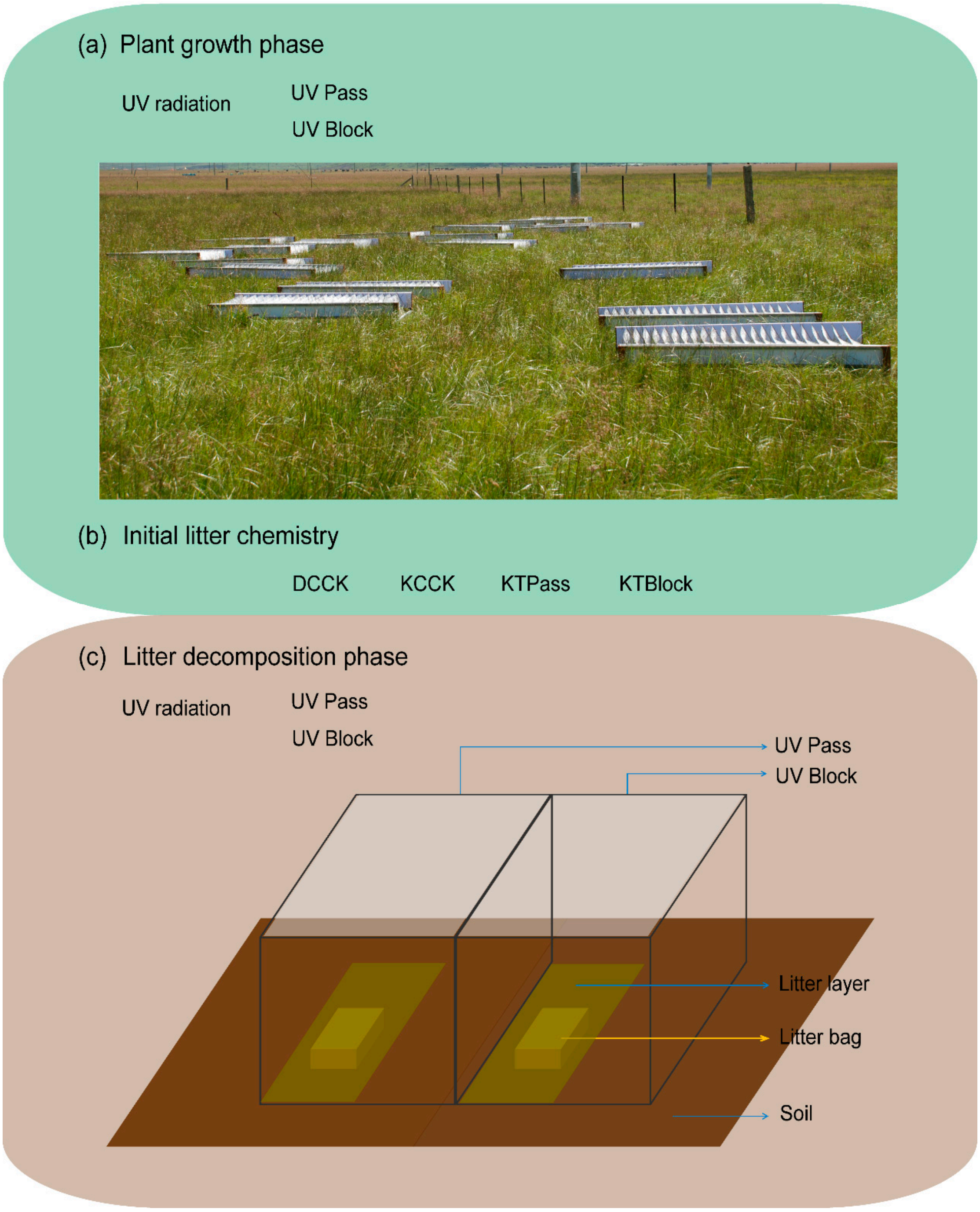

Figure S1 Litter decomposition in Zoige Alpine Meadow of the Qinghai-Tibetan

Plateau. (a) Changes in the chemical composition of organic carbon during the plant growth phase under ambient solar radiation and UV attenuation treatments. (b) Litter materials were classified into four distinct initial chemistries before decomposition: *Deschampsia caespitos*a (DCCK) and *Kobresia tibetica* (KTCK), both grown under ambient solar radiation (CK), and *K. tibetica* (KTPass and KTBlock), subjected to UV pass and UV block treatments, respectively. (c) The combined effects of UV radiation and soil microorganisms on litter decomposition.

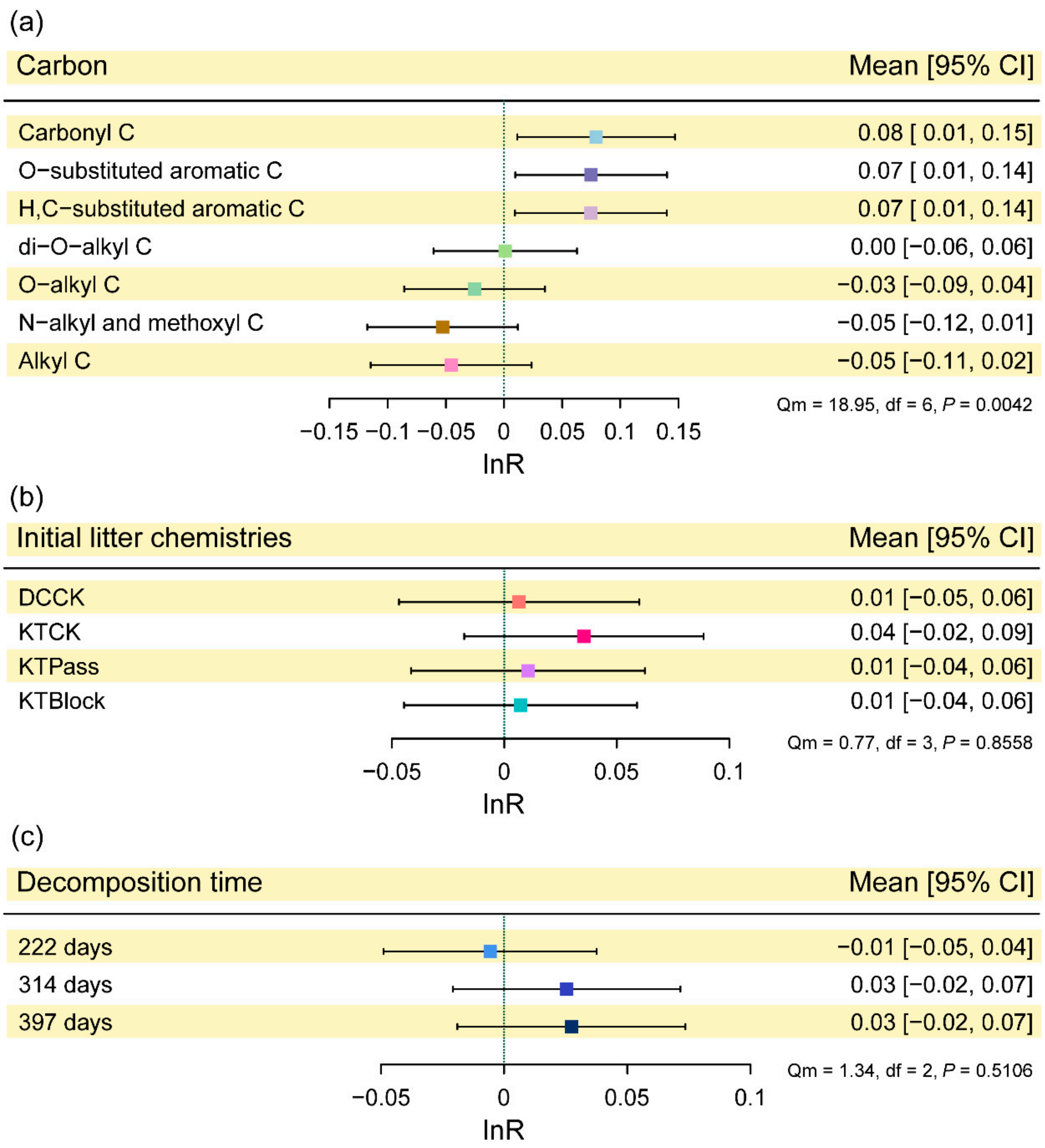

Figure S2 The effect of UV radiation on the relative abundance of litter organic carbon. Litter materials were classified into four distinct initial chemistries before decomposition: *Deschampsia caespitos*a (DCCK) and *Kobresia tibetica* (KTCK), both grown under ambient solar radiation (CK), and *K. tibetica* (KTPass and KTBlock) subjected to UV pass and UV block treatments, respectively. (a) Mean effect sizes of UV radiation on the relative abundance of organic carbon, partitioned into seven classes of organic carbon (±95% CI). (b) Mean effect sizes of UV radiation on the relative abundance of organic carbon, partitioned into four initial litter chemistries (±95% CI). (c) Mean effect sizes of UV radiation on the relative abundance of organic carbon, partitioned by three decomposition stages (±95% CI). lnR indicates the natural logarithm of the response ratio [±95% confidence intervals (CI)]. Q_m indicates variance explained by the categorical model. df indicates degrees of freedom.

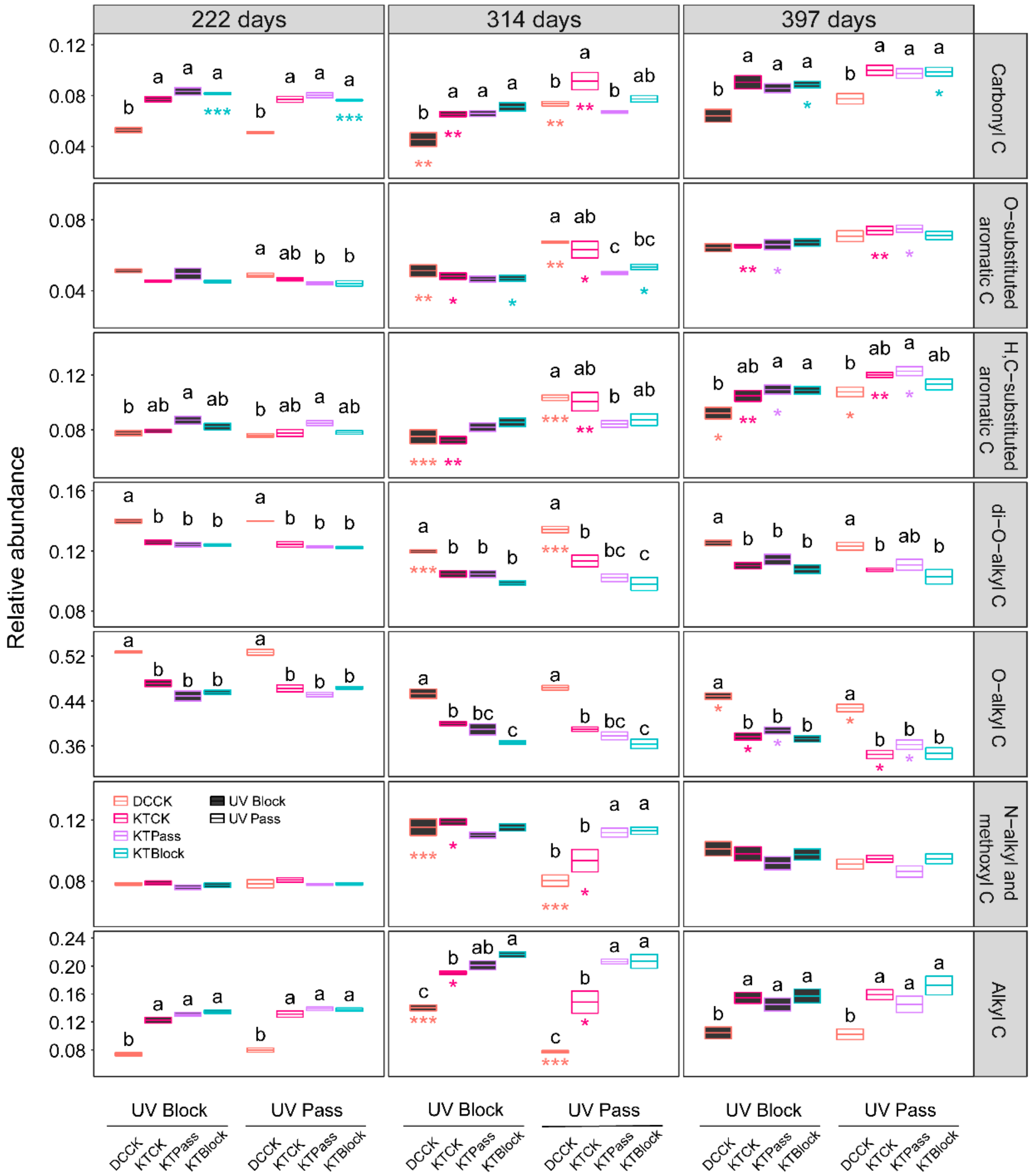

Figure S3 The relative abundance of chemical components of plant litter characterized by solid-state ^{13}C-CPMAS NMR spectra during decomposition. Litter materials were classified into four distinct initial chemistries before decomposition: *Deschampsia caespitos*a (DCCK) and *Kobresia tibetica* (KTCK), both grown under ambient solar radiation (CK), and *K. tibetica* (KTPass and KTBlock), subjected to UV pass and UV block treatments, respectively. Letters indicate significant differences in the relative abundance of organic carbon among initial litter chemistries under two UV radiation

treatments at different decomposition stages, respectively. Asterisks represent significant differences in the relative abundance of organic carbon for each initial litter chemistry between UV block and UV pass treatments across different decomposition stages (mean ± SE, n = 5, Tukey's HSD, and significance levels: *** 0.001, ** 0.01, *0.05).

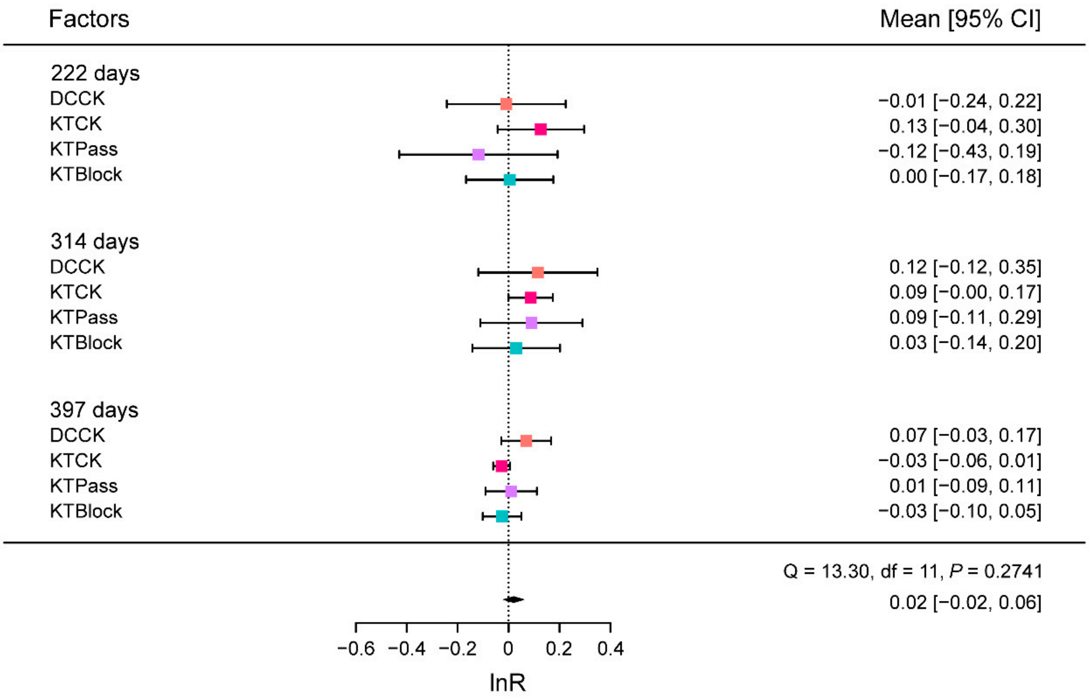

Figure S4 The effect of UV radiation on mass loss during litter decomposition. Litter materials were classified into four distinct initial chemistries before decomposition: *Deschampsia caespitos*a (DCCK) and *Kobresia tibetica* (KTCK), both grown under ambient solar radiation (CK), and *K. tibetica* (KTPass and KTBlock), subjected to UV pass and UV block treatments, respectively. lnR indicates the natural logarithm of the response ratio [±95% confidence intervals (CI)]. Q indicates total sample heterogeneity. df indicates degrees of freedom.

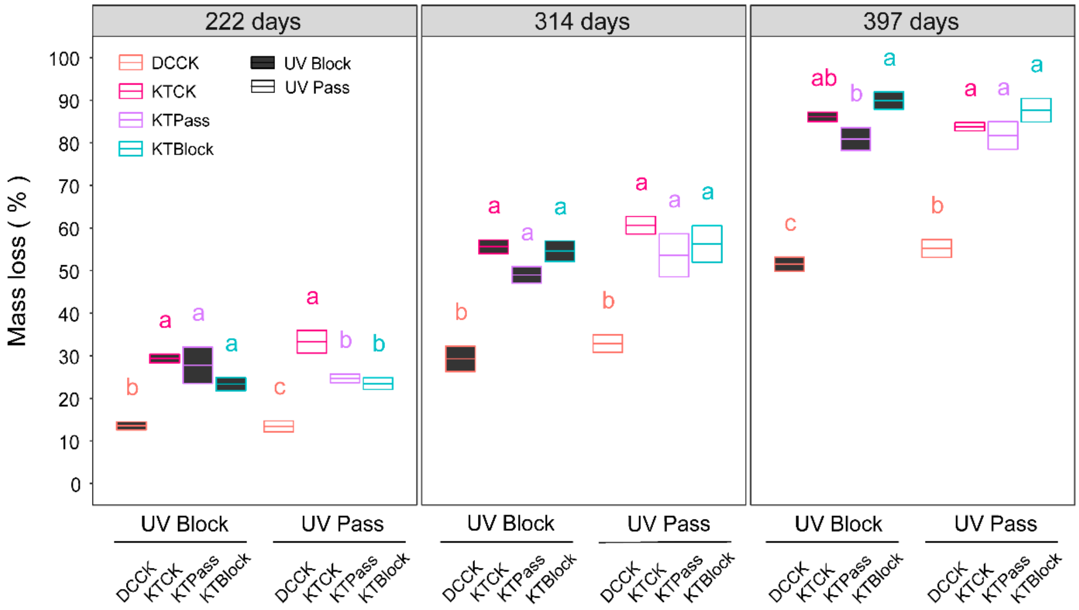

Figure S5 The mass loss of plant litter during decomposition. Litter materials were classified into four distinct initial chemistries before decomposition: *Deschampsia caespitos*a (DCCK) and *Kobresia tibetica* (KTCK), both grown under ambient solar radiation (CK), and *K. tibetica* (KTPass and KTBlock), subjected to UV pass and UV block treatments, respectively. Letters indicate significant differences in the mass loss among initial litter chemistries (DCCK, KTCK, KTPass, and KTBlock) under two UV treatments (UV Block and UV Pass) at different decomposition stages (mean ± SE, n = 5, Tukey's HSD, $P < 0.05$). There was no significant difference in the mass loss for each initial litter chemistry between UV block and UV pass treatments across different decomposition stages.

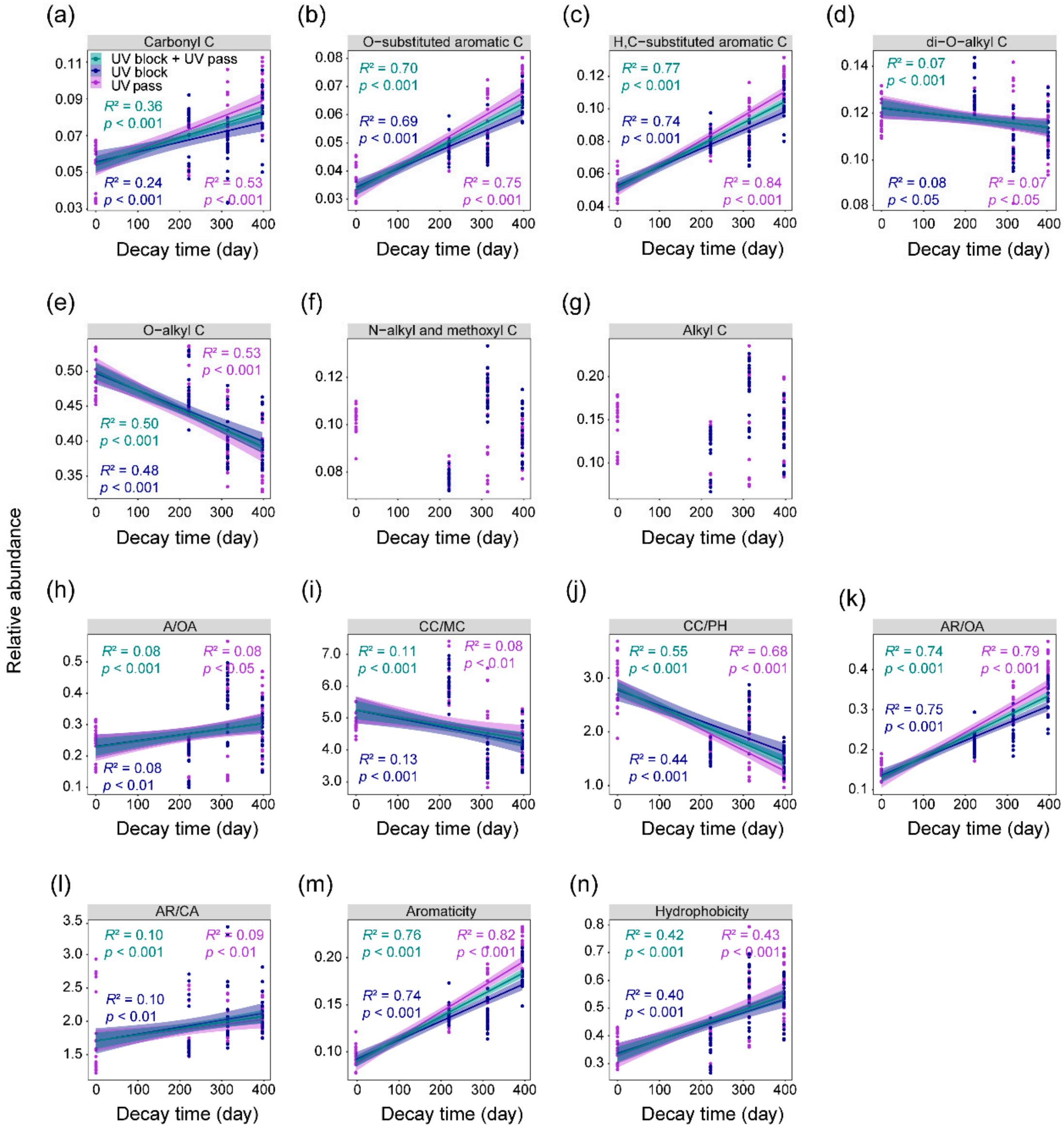

Figure S6 Relationships between litter chemical components and decay time during decomposition. Solid lines denote significant relationships ($P < 0.05$). The colors of the lines indicate UV block (blue), UV pass (purple), and general trends (green) throughout the decomposition process. Shading shows used to show the 95% confidence bands of the best-fit lines.

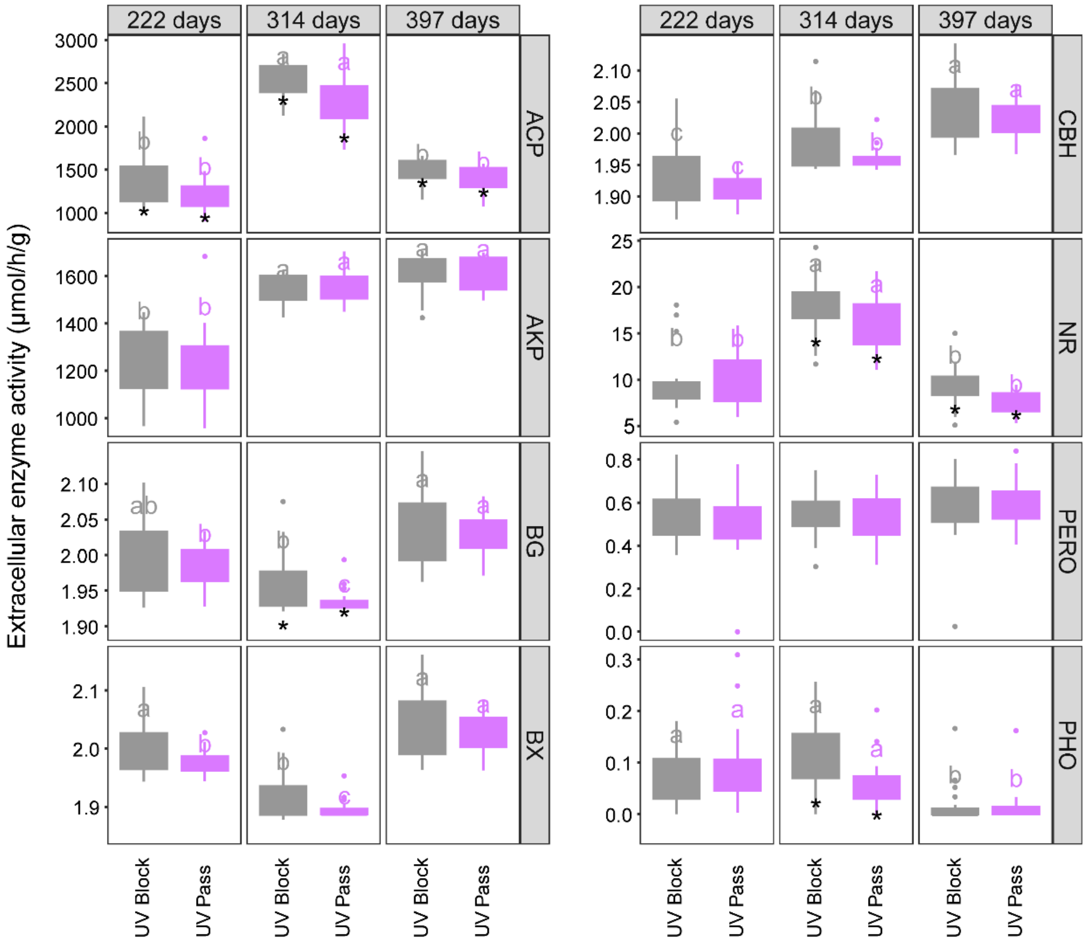

Figure S7 Extracellular enzyme activities in soil were measured after litter decomposition under UV block and UV pass treatments for 222, 314, and 397 days. Enzymes analyzed included cellobiohydrolase (CBH), β-1,4-glucosidase (BG), β-1,4-xylosidase (BX), phenol oxidase (PHO), peroxidase (PERO), acid phosphatase (ACP), alkaline phosphatase (ALP), and nitrate reductase (NR). Letters indicate significant differences in extracellular enzyme activity of soil microorganisms across three decomposition stages under two UV radiation treatments. Asterisks denote significant differences in extracellular enzyme activity of soil microorganisms between UV block and UV pass treatments at different decomposition stages (n = 5, Nemenyi test, significance level: * $P < 0.05$).

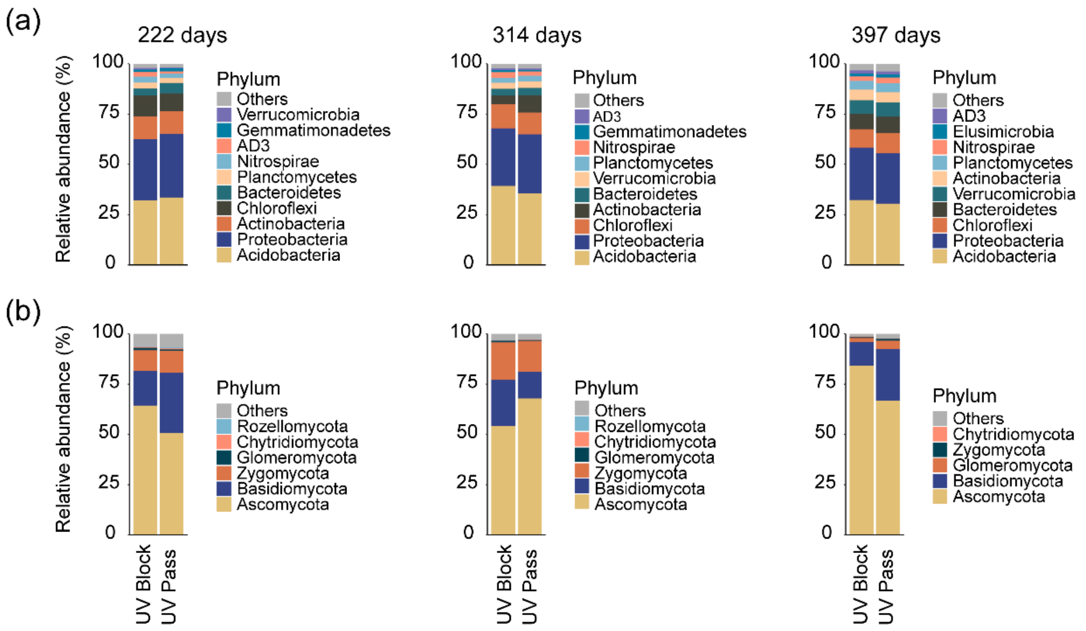

Figure S8 Microbial community composition of bacteria (a) and fungi (b) within the top 10 phyla across three stages of litter decomposition.

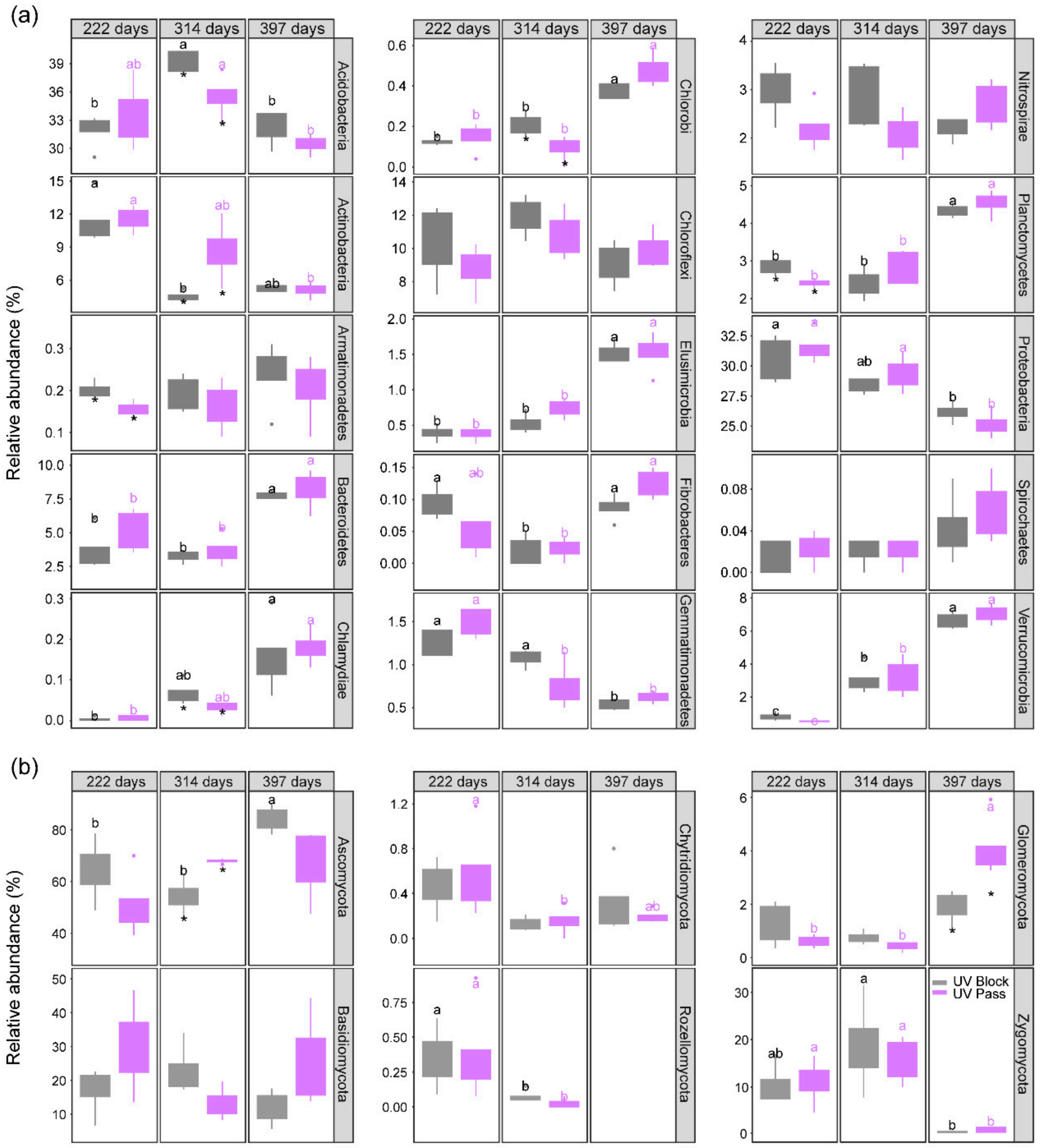

Figure S9 Relative abundance of bacteria (a) and fungi (b) during decomposition. Letters indicate significant differences in the relative abundance of soil microorganisms across three decomposition stages under two UV radiation treatment. Asterisks denote significant differences in the relative abundance of soil microorganisms between UV block and UV pass at different decomposition stages (n = 5, Tukey's HSD, significance level: * $P < 0.05$).

Table S1 Significance test of litter chemical complexity across initial chemistries of organic C during the growth phase.

Factors	Pairs	df	F	R^2	*P*	Sig
Litter	DCCK vs KTCK	1	80.3640	0.9095	**0.0090**	**
Litter	DCCK vs KTPass	1	47.9264	0.8570	**0.0120**	*
Litter	DCCK vs KTBlock	1	202.1823	0.9619	**0.0090**	**
Litter	KTCK vs KTPass	1	5.4357	0.4046	**0.0240**	*
Litter	KTCK vs KTBlock	1	12.0706	0.6014	**0.0120**	*
Litter	KTPass vs KTBlock	1	5.0176	0.3854	**0.0110**	*

The permutational multivariate analysis of variance (PERMANOVA) was performed on the basis of Bray–Curtis distance. Litter materials were classified into four distinct initial chemistries before decomposition: *Deschampsia caespitos*a (DCCK) and *Kobresia tibetica* (KTCK), both grown under ambient solar radiation (CK), and *K. tibetica* (KTPass and KTBlock), subjected to UV pass and UV block treatments, respectively. Early, mid, and late stages represent decay time at 222, 314, and 397 days. Significance levels are as follows: *** 0.001, ** 0.01, * 0.05.

Table S2 Significance test of litter chemical complexity across UV radiation treatments, initial litter chemistries, and decay time during the decomposition process.

	Levels	Pairs	df	F	R^2	*P*	Sig
	UV	Pass vs Block	1	3.4475	0.0284	**0.0330**	*
	Litter	DCCK vs KTCK	1	25.4081	0.3046	**0.0010**	***
	Litter	DCCK vs KTPass	1	29.6421	0.3382	**0.0010**	***
Factors	Litter	DCCK vs KTBlock	1	37.3352	0.3916	**0.0010**	***
	Litter	KTCK vs KTPass	1	0.2802	0.0048	0.7740	
	Litter	KTCK vs KTBlock	1	1.5932	0.0267	0.2000	
	Litter	KTPass vs KTBlock	1	0.6030	0.0103	0.5610	

	Time	early vs mid	1	42.3714	0.3520	**0.0010**	***
	Time	early vs late	1	91.9062	0.5409	**0.0010**	***
	Time	mid vs late	1	22.1154	0.2209	**0.0010**	***
UV	early	Pass vs Block	1	0.3660	0.0095	0.6210	
	mid	Pass vs Block	1	7.7948	0.1702	**0.0060**	**
	late	Pass vs Block	1	5.2865	0.1221	**0.0100**	**
Litter	early	DCCK vs KTCK	1	114.9298	0.8646	**0.0010**	***
	early	DCCK vs KTPass	1	150.0149	0.8929	**0.0010**	***
	early	DCCK vs KTBlock	1	204.1139	0.9190	**0.0010**	***
	early	KTCK vs KTPass	1	5.3459	0.2290	**0.0080**	**
	early	KTCK vs KTBlock	1	3.0357	0.1443	0.0670	.
	early	KTPass vs KTBlock	1	2.2714	0.1121	0.0900	.
	mid	DCCK vs KTCK	1	7.4421	0.2925	**0.0060**	**
	mid	DCCK vs KTPass	1	21.9146	0.5490	**0.0010**	***
	mid	DCCK vs KTBlock	1	26.8469	0.5986	**0.0010**	***
	mid	KTCK vs KTPass	1	4.0009	0.1818	0.0580	.
	mid	KTCK vs KTBlock	1	5.5602	0.2360	**0.0140**	*
	mid	KTPass vs KTBlock	1	3.7398	0.1720	**0.0220**	*
	late	DCCK vs KTCK	1	26.8707	0.5988	**0.0010**	***
	late	DCCK vs KTPass	1	17.5942	0.4943	**0.0010**	***
	late	DCCK vs KTBlock	1	27.1232	0.6011	**0.0010**	***
	late	KTCK vs KTPass	1	1.7083	0.0867	0.1480	
	late	KTCK vs KTBlock	1	0.3639	0.0198	0.7430	
	late	KTPass vs KTBlock	1	2.7838	0.1339	0.0630	.

The permutational multivariate analysis of variance (PERMANOVA) was performed on the basis of Bray–Curtis distance. Litter materials were classified into four distinct initial chemistries before decomposition:

*Deschampsia caespitos*a (DCCK) and *Kobresia tibetica* (KTCK), both grown under ambient solar radiation (CK), and *K. tibetica* (KTPass and KTBlock), subjected to UV pass and UV block treatments, respectively.

Early, mid, and late stages represent decay time at 222, 314, and 397 days. Significance levels are as follows: *** 0.001, ** 0.01, * 0.05.

Table S3 The relationship between the initial chemical components of litter organic C and lnR (mass loss), and lnR (k), respectively.

Initial chemical traits	lnR (mass loss)			lnR (k)		
	$F_{1,58}$	R^2	P	$F_{1,58}$	R^2	P
Carbonyl C	0.6908	0.0118	0.4093	0.7815	0.0133	0.3803
O-substituted aromatic C	0.1844	0.0032	0.6692	0.3295	0.0056	0.5682
H, C-substituted aromatic C	0.7756	0.0132	0.3821	0.4447	0.0076	0.5075
di-O-alkyl C	0.4302	0.0074	0.5145	0.7619	0.0130	0.3863
O-alkyl C	0.9257	0.0157	0.3400	1.0870	0.0184	0.3014
N-alkyl and methoxyl C	1.9280	0.0322	0.1702	1.3310	0.0224	0.2533
alkyl C	0.8400	0.0143	0.3632	1.1360	0.0192	0.2910
A/OA	0.9179	0.0156	0.3420	1.2340	0.0208	0.2712
CC/MC	0.1023	0.0018	0.7503	0.0000	0.0000	0.9961
MC/PH	0.0002	0.0000	0.9884	0.0481	0.0008	0.8271
AR/OA	0.5620	0.0096	0.4565	0.3564	0.0061	0.5528
AR/CA	0.3461	0.0059	0.5586	0.4447	0.0076	0.5075
Aromaticity	0.1876	0.0032	0.6666	0.0498	0.0009	0.8242
Hydrophobicity	1.1870	0.0201	0.2804	1.3960	0.0235	0.2421

Table S4 Relationships between litter chemical complexity and extracellular enzyme activities of soil microorganisms during the decomposition process.

Litter chemistry	Enzyme	*R*	*P*
Carbon	NR	0.15	**0.001***
Carbon	ACP	0.21	**0.001***
Carbon	AKP	0.34	**0.001***
Carbon	PHO	0.18	**0.001***
Carbon	PERO	0.05	0.13
Carbon	CBH	0.13	**0.002**
Carbon	BG	0.08	**0.005**
Carbon	BX	0.15	**0.002**

The enzymes analyzed included cellobiohydrolase (CBH), β-1,4-glucosidase (BG), β-1,4-xylosidase (BX), phenol oxidase (PHO), peroxidase (PERO), acid phosphatase (ACP), alkaline phosphatase (ALP), and nitrate reductase (NR). Significance levels are as follows: *** 0.001, ** 0.01, * 0.05.

Table S5 Litter chemical composition based on solid-state ^{13}C CPMAS NMR analyzed by three-way analysis of variance (ANOVA).

Factors	Carbonyl C			O-substituted aromatic C			H, C-substituted aromatic C			di-O-alkyl C			O-alkyl C			N-alkyl and methoxyl C			Alkyl C		
	df	F	*P*	df	F	*P*	df	F	*P*	df	F	*P*	df	F	*P*	df	F	*P*	df	F	*P*
UV	1	35.2416	**< 0.0001***	1	36.0990	**< 0.0001***	1	39.7260	**< 0.0001***	1	0.0283	0.8668	1	14.0196	**0.0003***	1	23.6392	**< 0.0001***	1	3.3729	0.0694
Litter	3	63.2734	**< 0.0001***	3	5.3298	**0.0019**	3	4.0548	**0.0093**	3	90.1442	**< 0.0001***	3	203.0303	**< 0.0001***	3	2.8787	**0.0400***	3	126.8563	**< 0.0001***
Time	2	70.4785	**< 0.0001***	2	246.1794	**< 0.0001***	2	187.0933	**< 0.0001***	2	131.5178	**< 0.0001***	2	484.8112	**< 0.0001***	2	159.5415	**< 0.0001***	2	114.5455	**< 0.0001***
UV × Litter	3	3.7732	**0.0131***	3	2.7954	**0.0444***	3	6.5976	**0.0004***	3	2.6838	0.0510	3	1.2745	0.2875	3	6.4972	**0.0005***	3	3.6548	**0.0152***
UV × Time	2	16.3120	**< 0.0001***	2	18.7228	**< 0.0001***	2	16.8400	**< 0.0001***	2	6.3779	**0.0025**	2	10.2959	**0.0001***	2	12.8573	**< 0.0001***	2	13.5800	**< 0.0001***
Litter × Time	6	4.4577	**0.0005***	6	4.8853	**0.0002***	6	4.7476	**0.0003***	6	3.8082	**0.0019**	6	4.3491	**0.0006***	6	4.5893	**0.0004***	6	7.5063	**< 0.0001***
UV × Litter × Time	6	2.5971	**0.0224***	6	1.6328	0.1464	6	2.8507	**0.0134***	6	1.4453	0.2055	6	0.4437	0.8478	6	4.5018	**0.0005***	6	2.9977	**0.0100**

Factors	A/OA			CC/MC			CC/PH			AR/OA			AR/CA			Aromaticity			Hydrophobicity		
	df	F	*P*	df	F	*P*	df	F	*P*	df	F	*P*	df	F	*P*	df	F	*P*	df	F	*P*
UV	1	0.0923	0.7619	1	5.8362	**0.0176***	1	34.2204	**< 0.0001*****	1	54.2233	**< 0.0001*****	1	6.1940	**0.0145***	1	46.6373	**< 0.0001*****	1	5.3768	**0.0225***
Litter	3	113.6942	**< 0.0001*****	3	44.1491	**< 0.0001*****	3	4.9587	**0.0030****	3	28.6964	**< 0.0001*****	3	143.4041	**< 0.0001*****	3	1.5742	0.2007	3	122.7652	**< 0.0001*****
Time	2	128.8414	**< 0.0001*****	2	358.5823	**< 0.0001*****	2	92.1724	**< 0.0001*****	2	375.9422	**< 0.0001*****	2	35.1241	**< 0.0001*****	2	235.1872	**< 0.0001*****	2	282.0295	**< 0.0001*****
UV × Litter	3	2.4930	0.0647	3	5.9231	**0.0009*****	3	4.6741	**0.0043****	3	3.3429	**0.0224***	3	4.1166	**0.0086****	3	5.4915	**0.0016****	3	1.2349	0.3014
UV × Time	2	8.8530	**0.0003*****	2	10.5195	**0.0001*****	2	23.5047	**< 0.0001*****	2	20.8396	**< 0.0001*****	2	2.6274	0.0775	2	20.9453	**< 0.0001*****	2	8.7311	**0.0003*****
Litter × Time	6	8.8038	**< 0.0001*****	6	4.5634	**0.0004*****	6	4.8945	**0.0002*****	6	6.1412	**< 0.0001*****	6	5.2798	**0.0001*****	6	5.5580	**0.0001*****	6	7.4938	**< 0.0001*****
UV × Litter × Time	6	1.8486	0.0977	6	3.7954	**0.0020****	6	2.8401	**0.0137***	6	1.1030	0.3663	6	2.3604	**0.0360***	6	2.6162	**0.0216***	6	0.8275	0.5515

Degree of freedom, F-value and *P* statistic are represented by df, F and *P* respectively in the table. The UV radiation, initial litter chemistries, and decomposition time are represented by UV, Litter, and Time, respectively.

Significance levels are as follows: *** 0.001, ** 0.01, * 0.05.

Table S6 Mass loss of plant litter analyzed by three-way analysis of variance (ANOVA).

Factors	df	F	*P*
UV	1	1.6810	0.1979
Litter	3	134.9306	**< 0.0001*****
Time	2	940.8081	**< 0.0001*****
UV × Litter	3	0.3710	0.7741
UV × Time	2	1.4393	0.2422
Litter × Time	6	9.1979	**< 0.0001*****
UV × Litter × Time	6	0.5948	0.7338

Degree of freedom, F-value and *P* statistic are represented by df, F and *P* respectively in the table. The UV radiation, initial litter chemistries, and decomposition time are represented by UV, Litter, and time, respectively.

Significance levels are as follows: *** 0.001 ** 0.01 * 0.05.

Reference

1. Bonanomi, G.; Incerti, G.; Giannino, F.; Mingo, A.; Lanzotti, V.; Mazzoleni, S. Litter quality assessed by solid state C-13 NMR spectroscopy predicts decay rate better than C/N and Lignin/N ratios. *Soil Biology & Biochemistry* **2013**, *56*, 40-48, doi:10.1016/j.soilbio.2012.03.003.
2. Xu, Y.; Fan, J.; Ding, W.; Gunina, A.; Chen, Z.; Bol, R.; Luo, J.; Bolan, N. Characterization of organic carbon in decomposing litter exposed to nitrogen and sulfur additions: Links to microbial community composition and activity. *Geoderma* **2017**, *286*, 116-124, doi:10.1016/j.geoderma.2016.10.032.
3. Chavez-Vergara, B.; Merino, A.; Vazquez-Marrufo, G.; Garcia-Oliva, F. Organic matter dynamics and microbial activity during decomposition of forest floor under two native neotropical oak species in a temperate deciduous forest in Mexico. *Geoderma* **2014**, *235*, 133-145, doi:10.1016/j.geoderma.2014.07.005.
4. De Marco, A.; Spaccini, R.; Vittozzi, P.; Esposito, F.; Berg, B.; De Santo, A.V. Decomposition of black locust and black pine leaf litter in two coeval forest stands on Mount Vesuvius and dynamics of organic components assessed through proximate analysis and NMR spectroscopy. *Soil Biology & Biochemistry* **2012**, *51*, 1-15, doi:10.1016/j.soilbio.2012.03.025.
